# Mesenchymal Stem Cells (MSC) Prevented the Progression of Renovascular Hypertension, Improved Renal Function and Architecture

**DOI:** 10.1371/journal.pone.0078464

**Published:** 2013-11-04

**Authors:** Elizabeth B. Oliveira-Sales, Edgar Maquigussa, Patricia Semedo, Luciana G. Pereira, Vanessa M. Ferreira, Niels O. Câmara, Cassia T. Bergamaschi, Ruy R. Campos, Mirian A. Boim

**Affiliations:** 1 Departament of Medicine, Renal Division, Federal University of Sao Paulo, Sao Paulo, Brazil; 2 Cardiovascular Division – Department of Physiology, Federal University of Sao Paulo, Sao Paulo, Brazil; 3 Immunology Department, University of Sao Paulo, Sao Paulo, Brazil; University of Sao Paulo – USP, Brazil

## Abstract

Renovascular hypertension induced by 2 Kidney-1 Clip (2K-1C) is a renin-angiotensin-system (RAS)-dependent model, leading to renal vascular rarefaction and renal failure. RAS inhibitors are not able to reduce arterial pressure (AP) and/or preserve the renal function, and thus, alternative therapies are needed. Three weeks after left renal artery occlusion, fluorescently tagged mesenchymal stem cells (MSC) (2×10^5^ cells/animal) were injected weekly into the tail vein in 2K-1C hypertensive rats. Flow cytometry showed labeled MSC in the cortex and medulla of the clipped kidney. MSC prevented a further increase in the AP, significantly reduced proteinuria and decreased sympathetic hyperactivity in 2K-1C rats. Renal function parameters were unchanged, except for an increase in urinary volume observed in 2K-1C rats, which was not corrected by MSC. The treatment improved the morphology and decreased the fibrotic areas in the clipped kidney and also significantly reduced renal vascular rarefaction typical of 2K-1C model. Expression levels of IL-1β, TNF-α angiotensinogen, ACE, and Ang II receptor AT_1_ were elevated, whereas AT_2_ levels were decreased in the medulla of the clipped kidney. MSC normalized these expression levels. In conclusion, MSC therapy in the 2K-1C model (i) prevented the progressive increase of AP, (ii) improved renal morphology and microvascular rarefaction, (iii) reduced fibrosis, proteinuria and inflammatory cytokines, (iv) suppressed the intrarenal RAS, iv) decreased sympathetic hyperactivity in anesthetized animals and v) MSC were detected at the CNS suggesting that the cells crossed the blood-brain barrier. This therapy may be a promising strategy to treat renovascular hypertension and its renal consequences in the near future.

## Introduction

Renovascular hypertension induced by 2 Kidney-1 Clip (2K-1C) is a renin-angiotensin-system (RAS)-dependent model, leading to intrarenal vascular rarefaction and renal failure [1]–[3]. Previous studies showed that the increase in circulating levels of Angiotensin II (Ang II) in the 2K-1C model produces higher appetite and sodium retention, blood volume expansion and increased systemic vascular resistance, as well as increased production of aldosterone and antidiuretic hormone, activation of the sympathetic nervous system (SNS), proliferation, fibrosis and renal injury, all of which lead to increased arterial pressure (AP) [4]–[8]. Indeed, Ang II activates the NAD (P) H oxidases, which produce superoxide, alter the bioavailability of nitric oxide and consequently cause greater oxidative stress and inflammation, contributing to endothelial dysfunction and vascular remodeling [9]–[12].

The control of renovascular hypertension is problematic. Many antihypertensive drugs, including the RAS blockers, are not always effective, and 60% of patients are refractory to the treatment, indicating that new approaches are needed. Recent studies have shown that stem cells have emerged as a new therapy for many diseases [13]–[17]. Hematopoietic stem cells and mesenchymal stem (stromal) cells (MSC) can release growth factors and cytokines, which may act by a paracrine action on neighboring cells, stimulating proliferation, survival and angiogenesis by inhibiting apoptosis, fibrosis and oxidative stress [18]. VEGF, HGF, IGF-1 and adrenomedullin are among the growth factors described, and when they are administered intravenously all of these factors have antihypertensive actions, emphasizing the role of stem cells in reducing systemic AP due to paracrine effects on blood vessels [18]. However, the mechanisms underlying the effects of MSC in renovascular hypertension are poorly understood.

Therefore, we determined whether treatment with MSC can improve the renovascular hypertension, modify RAS components, restore the renal architecture and microvascular rarefaction in the ischemic kidney, improve renal function and decrease sympathetic nerve activity (SNA) in the model 2K-1C. Furthermore, the tracking of MSC cells at the kidney and Central Nervous System (CNS) were investigated considering that these two regions are involved in the generation and maintenance of renovascular hypertension.

## Materials and Methods

### Experimental Protocol

All experimental procedures were approved by the Ethics in Research Committee of the Federal University of Sao Paulo – School of Medicine (CEP 1017/10). Male Wistar rats (150–180 g) from the animal facility of the Federal University of Sao Paulo, Sao Paulo, Brazil were housed in cages, with free access to rat chow and tap water, and maintained in a temperature-controlled environment (23°C) on a 12-h light/dark cycle.

### Renovascular Hypertension Model

The animals were anesthetized with intraperitoneal (I.P.) injections of ketamine and xylazine (40 and 20 mg/kg, respectively) (Vetbrands, Jacareí, SP, Brazil), and the left renal artery was partially obstructed with a 0.2 mm silver clip^4^. The control animals (SHAM) were submitted to the same surgical procedure but with no renal artery occlusion.

### MSC Isolation and Characterization

**Figure 1:**
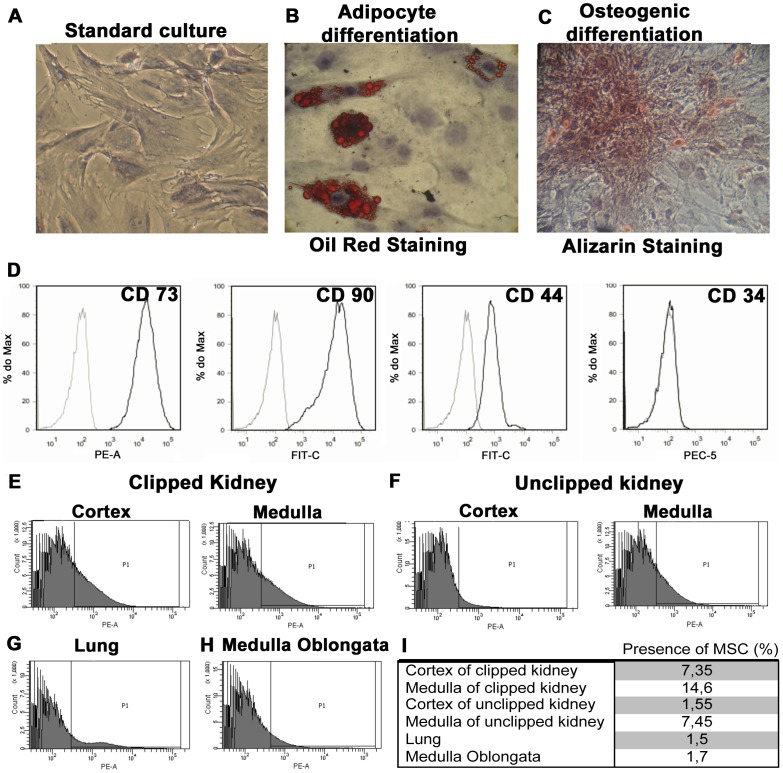
Adipocyte and osteogenic differentiation and MSC tracking assay. Standard culture (**A**), differentiated adipocytes containing lipid droplets visualized by Oil Red coloration (**B**), and osteocytes exhibiting calcium deposition (**C**), demonstrated by Alizarin coloration. Magnification: 100x. (**D**) MSC immunophenotyping. MSCs were isolated and labeled for CD73, CD90, CD44 and CD34. Labeled MSC with fluorescent marker detected by cytometry of 2K-1C animals (n = 2) and 2K-1C + MSC (n = 2) in the cortex and medulla of the clipped kidney (**E**), unclipped kidney (**F**), lung (**G**) and medulla oblongata (**H**). Percentage of labeled cells found in the cortex and medulla of clipped and unclipped kidney, lung and medulla oblongata, analyzed by FACS (**I**).

MSC were isolated from the tibias and femurs of male Wistar rats (150 to 180 g) [19]. Flushed cells were subjected to Histopaque (Sigma, Aldrich, St Louis, MO, USA) density gradient separation, and the mononuclear fraction was harvested. This fraction was cultured on plastic dishes in low-glucose DMEM (Gibco, NY, USA) supplemented with 10% FCS (Emcare, Campinas, Brazil) until the cells reached 70–80% confluence. Cells were detached with trypsin and subsequently cultured until the 7^th^ passage. At the 2^nd^ passage, cells were subjected to osteogenic and adipocyte differentiation assays. For adipocyte assay, cells were incubated with DMEM containing low-glucose and 10% FBS supplemented with dexamethasone (1 µM), IBMX (0.5 mM), insulin (10 µg/mL), and indomethacin (100 µM) for 3 days, and then culture medium was changed to DMEM containing only insulin (20 µg/mL) for 21 days. Then, the differentiated adipocytes were stained with oil red **(** **Figure 1B** **)**. For osteogenic differentiation, MSC were incubated in DMEM containing low glucose and 10% FCS supplemented with dexamethasone (0.1 µM), ascorbic acid (0.2 mM), and beta glycerol phosphate (10 mM) for 28 days and stained with alizarin **(** **Figure 1C** **)**. All reagents were purchased from Sigma, St. Louis, USA. For animal administration, MSC were obtained from passage 3 to 7. Immunophenotype assays were performed with CD73, CD90, CD44 (all from BD Bioscience Pharmingen, San Diego, CA, USA) and CD34 (Santa Cruz Biotechnology, Inc, CA, USA) every time before administration at animals. The cells were analyzed at FACS (FACS Calibur, BD). They were positive for CD73 (99,8%), CD90 (99,9%) and CD44 (97,6%) and negative for CD34 (1,7%) **(** **Figure 1D** **)**.

### MSC Treatment

Animals were divided in the following groups: SHAM (n = 5), 2K-1C (n = 8) and 2K-1C+MSC-treated (n = 7). Three weeks after left renal artery occlusion or SHAM occlusion, MSC (2×10^5^ cells/animal) were injected weekly into the tail vein (two administrations at the 3^rd^ and 5^th^ weeks). Animals were sacrificed 6 weeks after clipping.

### Cell Labeling for Tracking Assay

The MSC were incubated with Qtracker 585 nm (Invitrogen, CA, USA) as recommended by the manufacturer, and then endovenously injected in three animals of the MSC-treated group at the 3^rd^ and 5^th^ weeks after surgery. These animals were sacrificed at the 6^th^ week after MSC administration. Untreated animals were used as controls. Cortical and medullary sections of the kidney, medulla oblongata and lungs were passed through a 70 µM cell strainer (Becton Dickinson, Franklin Lakes, NJ, USA). The cells were washed twice with cold PBS (200 g, 5 minutes), subjected to flow cytometry (FACSCanto; Becton Dickinson, Franklin Lakes, NJ, USA) and visualized through a PE filter set.

### Recording Blood Pressure and Analysis of the Renal Sympathetic Nerve Activity (RSNA) in Urethane Anesthetized Rats

The systolic arterial pressure (SBP) was analyzed every week after the clipping by tail-cuff plethysmography (Narco Bio-systems, INC., Houston, TX, USA). In separated series of experiments, others animals were used to SNA analysis. Animals were divided in the following groups: SHAM (n = 6), 2K-1C (n = 6) and 2K-1C+MSC-treated (n = 6). After 6 weeks, the rats was slowly anesthetized with urethane (1.2 g/kg, i.v.) (Sigma-Aldrich Co, St Louis, MO, USA) to avoid any change in basal cardiovascular conditions. The left renal nerve was retroperitoneally exposed and placed on bipolar silver electrodes. When the conditions for nerve recording had been established, the nerve and electrode were covered with paraffin oil. The signal from the renal nerve was displayed on an oscilloscope (Tektronix, TDS 220, USA), and the nerve activity was amplified (gain 20 K, Neurolog, Digitimer, UK), filtered by a band-pass filter (100–1000 Hz), and collected for display and later analysis using a PowerLab data acquisition system (ADInstruments, Australia). At the end of the experiments, the background noise level was determined by hexamethonium bromide administration (30 mg/kg, i.v.) (Sigma-Aldrich Co, St Louis, MO, USA). The neural activity was analyzed offline using the appropriate software (Spike Histogram, ADInstruments - Australia), the raw nerve signal was passed through a spike discriminator (PowerLab ADInstruments - Australia) to remove background noise, and then the total nerve activity expressed in spikes per second (spikes/s). The basal RSNA was expressed as spikes/s over a period of 60 s. Only experiments in which the level of background noise was confirmed at the end of the experiments following hexamethonium administration and terminal anesthesia were included in this report.

### Assessment of Renal Function

Six weeks after the clipping, the animals were housed in metabolic cages for 24-hr urine collection, and then the animals were anesthetized with ketamine and xylazine and aorta blood samples were collected. Plasma and urinary concentrations of sodium and potassium (Flame Photometer B462 Micronal, Sao Paulo, Brazil), creatinine (Labtest Kit, Lagoa Santa, Brazil) and urinary protein excretion (Sensiprot, Labtest Diagnostics, Lagoa Santa, Brazil) were determined.

### Kidney Morphology and Immunohistochemical Staining

Kidneys were removed and fixed with 10% buffered formaldehyde for 24 hr, washed with 70% ethanol for 24 hr and then embedded in paraffin. Sections were cut with a thickness of 4 mm and stained with Hematoxylin-Eosin *(H&E)* and Picrosirius red. Fibrotic areas were measured using the Image Tool program at a magnification of 20x. A blinded reviewer compared the 2K-1C kidneys, treated or not, with the SHAM group.

For immunohistochemical procedures, the kidney slices were deparaffinized and rehydrated. To expose the antigens, kidney sections were boiled in a target retrieval solution (TRIS buffer pH 9.0) for 30 min. Endogenous peroxidase activity was blocked with 3% H_2_O_2_ for 15 min at room temperature. Nonspecific binding was prevented by incubating the sections with a protein blocker (Dako, CA, EUA). Sections were incubated overnight at 4° C with a primary mouse anti-aminopeptidase P (JG-12) monoclonal antibody for endothelial cells (1:1000, eBioscience, San Diego, CA USA). After washing with TBS, the sections were incubated with horseradish peroxidase-conjugated polymer (Dako, CA, EUA) for 30 min at room temperature. Slides were rinsed with PBS, and the sites of antibody-antigen reactions were visualized with 3,3’-diaminobenzidine (Dako, CA, EUA). The sections were lightly counterstained with hematoxylin. Analyses were carried out with a light microscopy (Leica Imaging Systems) using x20 magnification, and stained proteins were quantified using Corel Photo-Paint 12 and UTHSCSA – ImageTool software. The picture analysis was done in two phases, being the first phase measure the tissue area subtracting blank areas on picture, and the second phase was done calculating the percentage of staining present on first phase picture.

### Protein Expression Levels

Protein levels of angiotensinogen (AGTN), renin, angiotensin-converting enzyme (ACE), Ang II receptors types 1 (AT_1_) and 2 (AT_2_) were determined in the renal medulla of the clipped kidneys by western blot. Kidney fragments were homogenized (homogenizer, Polytron, Switzerland) in ice-cold buffer (50 mM TRIS, 150 mM NaCl, 1,0% nonidet P-40, 0.5% Deoxicolato de Sódio, 0.1% SDS – pH  =  8.0) containing a proteases inhibitor pool (Sigma Chemical CO, St. Louis, EUA). Total protein was measured by a modified Lowry method (Bio-Rad DC protein assay reagents, Bio-Rad, Richmond, California, USA). Protein samples (50 µg) were separated by size using SDS-PAGE electrophoresis and electroblotted onto nitrocellulose membrane (Amersham Pharmacia Biotech, Piscataway, NJ, USA). The membrane blots were probed with the following primary antibodies: Anti-AGTN 1∶5000 (Abcam, Cambridge, MA, USA), Anti-ECA 1∶5000 (Abcam, Cambridge, MA, USA), Anti-renin 1∶100 (Santa Cruz Biotechnology, Inc, CA, USA), Anti-AT_1_ 1∶50 000 and Anti-AT_2_ 1∶4000 (Proteimax, Cotia, Brazil), all of which were followed by HRP-conjugated second antibodies. The bands were visualized using Immobilon Western HRP Substrate (Millipore Corporation, Billerica, EUA). The bands were quantified using Luminescent Image Analyzer-LAS 4000 and the Image Gauge V3.1 software (Fuji Photo Film CO, Japan).

### Gene Expression Levels

Total RNA was purified from the whole kidneys by the phenol and guanidine isothiocyanate-cesium chloride method using an appropriate kit (TRIzol; Gibco BRL, Rockland, MD., USA), following the manufacturer’s instructions. Two µg of total RNA were treated with DNase (RQ1 RNase-free DNase; Promega, Madison, WI, USA), to avoid genomic DNA contamination, and reverse transcribed into cDNA by the addition of a mixture containing 0.5 mg/ml of oligo(dT), 10 mM of DTT, 0.5 mM of deoxynucleoside triphosphates (Amersham Pharmacia Biotech, Uppsala, Sweden) and 200 U of reverse transcriptase enzyme (SuperScript RT; Gibco BRL). The mRNA expression levels were estimated by quantitative real-time PCR (Gene-Amp 5700; Applied Biosystems, Foster City, CA, USA) using the following specific primers for each molecule (forward and reverse, respectively): β-actin (5′ CCTCTATGCCAACACAGTGC 3′ and 5′ACATCT-GCTGGAAGGTGGAC 3′), TNF-α (5' CAG AGC AAT GAC TCC AAA GTA GAC CT 3' and 5' CAG ACC CTC ACA CTC AGA TCA TCT T 3'), interleukin (IL)-10 (5' GGT TTT CCA AGG AGT TGC TCC 3' and 5' ATT GAA CCA CCC GGC ATC TAC 3') and IL-1β (5' TCT-CAA-GCA-GAG-CAC-AGA-CC3' and 5' GAC-AAA-GGC-TTC-CCC-TGG-AG 3'). Relative expression of the target genes was normalized with the values obtained for the β-actin housekeeping gene. PCR product accumulation was monitored using SYBR Green I intercalating dye (Molecular Probes, Eugene, OR, USA), which exhibits higher fluorescence upon binding of double-stranded DNA.

### Statistical Analysis

Results are presented as the Mean ± Standard Error. Data were evaluated using one-way ANOVA followed by the Tukey’s post-test method or Newman Keuls’s when appropriate and we using the two-way ANOVA followed by the Bonferroni’s post-test to analyze the blood pressure. Statistical significance was defined as *P*<0.05.

## Results

### MSC Localization in Kidney, Medulla Oblongata and Lung after I.V. Administration

Firstly, we focused on tracking MSC at the cortex and medulla of the clipped and unclipped kidneys, lungs and medulla oblongata. MSC labeled with Qtracker were found mainly in the cortex and medulla of the clipped kidneys and also in the medulla of the unclipped kidneys. Cells were also detected in the lungs and medulla oblongata, but to a lesser extent **(** **Figure 1D-G** **)**.

### Cell Therapy Prevented the Progressive Increase of Blood Pressure and Reduced the Renal Sympathetic Nerve Activity

**Figure 2:**
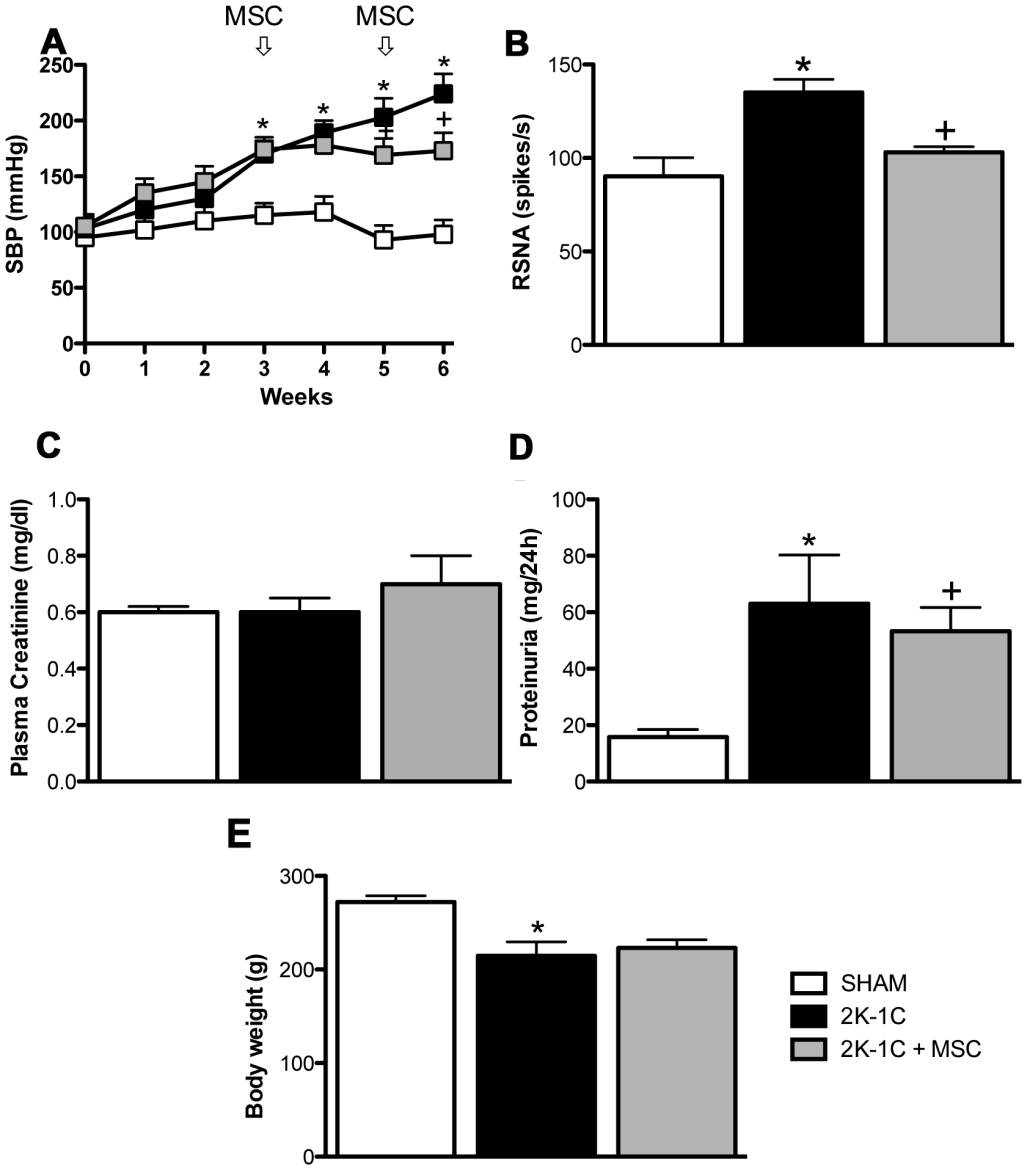
Systolic blood pressure (SBP), renal sympathetic nerve activity (RSNA) and renal functional parameters. (**A**) SBP expressed as the mean ± SEM. *P<0.05 vs. SHAM. ^+^ *P<0.05 vs. 2K-1C* (2-way ANOVA followed by the Bonferroni’s post-test). RSNA (**B**), plasma creatinine (**C**), proteinuria (**D**) and body weight (**E**) of SHAM animals (n = 5), 2K-1C animals (n = 7) and 2K-1C + MSC animals (n = 7). *P<0.05 vs. SHAM. ^+^ *P<0.05 vs. 2K-1C.* (One-way ANOVA followed by the Newman Keuls’s post-test).

The SBP was initially recorded by tail-cuff plethysmography every week before administration of MSC and for an additional 3 weeks after cell therapy. In all groups, baseline levels of SBP were similar **(** **Figure 2A** **)**. SBP increased gradually for 2 weeks after renal artery clipping and by the 3 ^rd^ week, SBP was significantly elevated in the 2K-1C rats compared with the SHAM group. MSC administration in the 2K-1C was performed at the end of the third and fifth weeks. Although SBP was not reduced by MSC treatment, it prevented the increase in SBP observed in the untreated 2K-1C rats **(** **Figure 2A** **)**. Indeed, we observed sympathetic hyperactivity in anesthetized animals, when we measured the RSNA 6 weeks after clipping and the MSC treatment reduced significantly in the hypertensive rats **(** **Figure 2B** **).**

### Renal Functional Parameters

There was no significant change in plasma creatinine levels among groups **(** **Figure 2C** **).** However, hypertensive animals developed more proteinuria when compared with the SHAM animals (15±3 vs. 80±19 mg/24 h, p<0.05) and MSC treatment significantly reduced proteinuria in 2K-1C animals **(** **Figure 2D** **)**. As show in **Table 1**, the plasma Na^+^ concentration was similar among groups. Serum K^+^ levels decreased 6 weeks after clipping, and MSC treatment caused a rise in serum K^+^ that reached values close to the SHAM group. Finally, 2K-1C animals presented with a significant increase in 24 hr urinary volume, with a decrease in urinary excretion of Na^+^, indicating a deficient mechanism of urinary concentration capacity. MSC treatment improves the urinary excretion of Na^+^, but did not correct the urinary volume. The hypertensive animals showed decreased body weight when to compare to the SHAM rats (214±15 vs. 272±6 g), however the MSC treatment didn’t change these values (223±8 vs. 295±5 g) **(** **Figure 2E** **).**

**Table 1:** Plasma and urinary parameters.

	SHAM	2K-1C	2K-1C + MSC
**Plasma values**			
Na^+^ (mEq/l)	141,0±0,5	141,0±1,0	142,0±2,4
K^+^ (mEq/l)	5,5±0,2	5,0±0,3^a^	5,3±0,2^b^
**Urinary values**			
Na^+^ (mEq/24 h)	2,4±0,1	1,0±0,3^a^	2,4±0,3^b^
K^+^ (mEq/24 h)	4,8±0,1	5,7±0,3 a	3,2±1,6
Total urinary volume (ml)	12,0±0,9	28,0±4,4 a	33,0±8,5

Values are expressed as the mean ± SE.

SHAM (n = 5), SHAM+MSC-treated (n = 2), 2K-1C (n = 8) and 2K-1C+MSC-treated (n = 7).

a *P<0.05 vs. SHAM group*. ^b^ *P<0.05 vs. 2K-1C group*.

(One-Way ANOVA following by Newman- Keuls’s pos-test).

### Morphology, Fibrosis and Microvascular Rarefaction of the Clipped Kidneys were Ameliorated after Treatment with MSC

**Figure 3:**
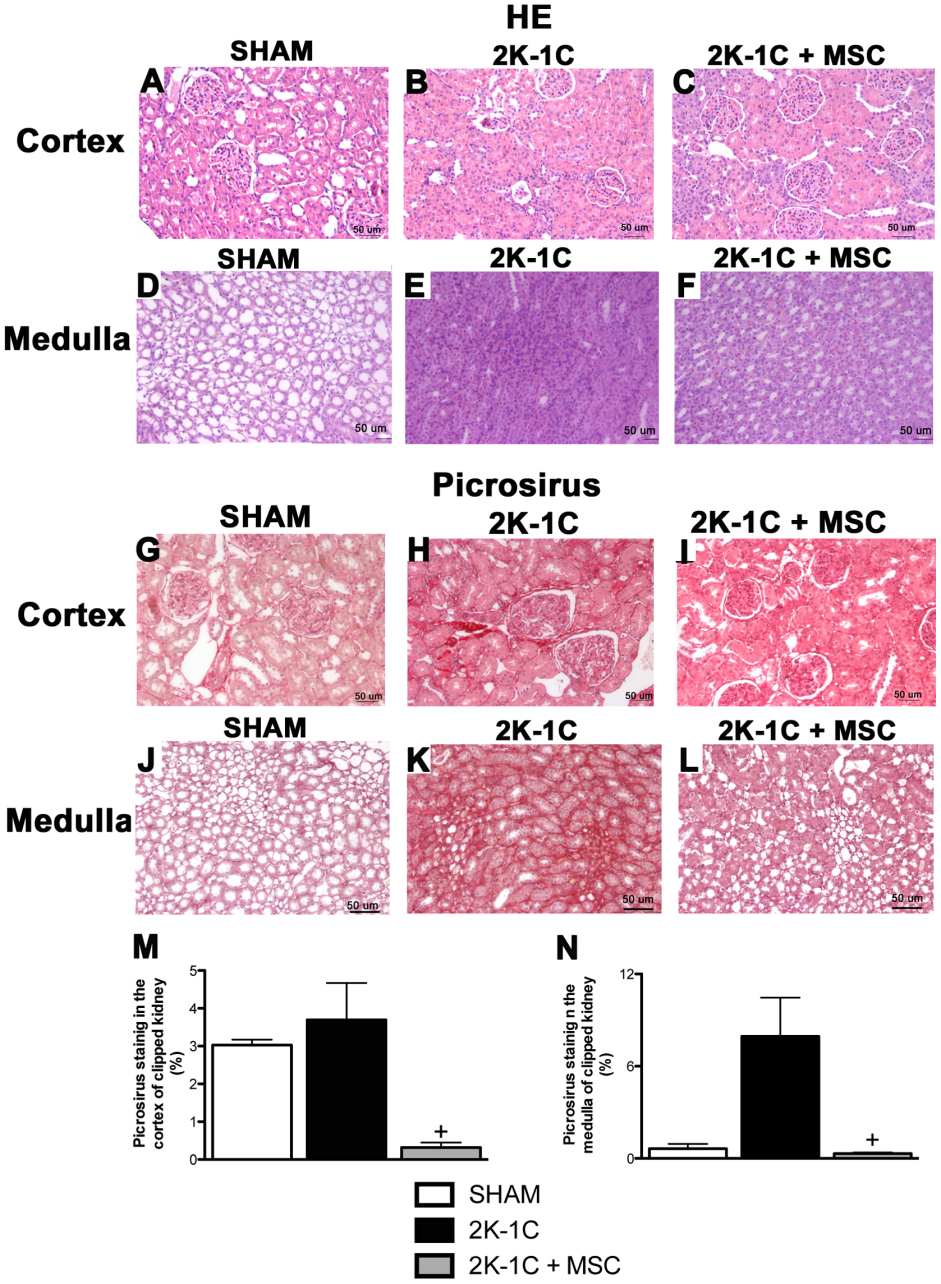
Morphology and fibrosis of the kidney. Hematoxylin-Eosin *(H&E)* (**A-F**) and Picrosirius red (**G-L**) staining (20x) of a renal cortex and medulla of the clipped kidneys of SHAM, 2K-1C and 2K-1C + MSC animals. Fibrotic area was measured using the Image Tool program (**M and N**). ^*^ *P<0.05 vs. 2K-1C.* (One-way ANOVA followed by the Newman Keuls’s post-test.

By HE staining, the 2K-1C rats showed glomerular atrophy, and the proximal tubular cells were severely damaged when compared with the SHAM rats. The damage included widespread loss of brush border, peritubular capillary congestion and edema in the cortex and medulla of the clipped kidneys **(** **Figure 3** **, panels A, B, D and E)**. After the treatment with MSC there was an improvement in the morphology of the clipped kidneys in both the renal cortex **(** **Figure 3C** **)** and medulla **(** **Figure 3F** **)**.

The Picrosirius red staining revealed the presence of collagen in the cortex, mainly in the medulla of the clipped kidneys of 2K-1C rats, indicating the presence of a fibrogenesis process when compared with the SHAM group **(** **Figure 3** **, panels G, H, J and K)**. The 2K-1C-MSC-treated animals showed an impressive improvement in the medullar tissue with much less collagen staining, which was similar to the SHAM animals **(** **Figure 3** **, panels I and L).** Picrossirus-stained areas were quantified and results are shown in **Panels M and N of** **Figure 3** **.**

**Figure 4:**
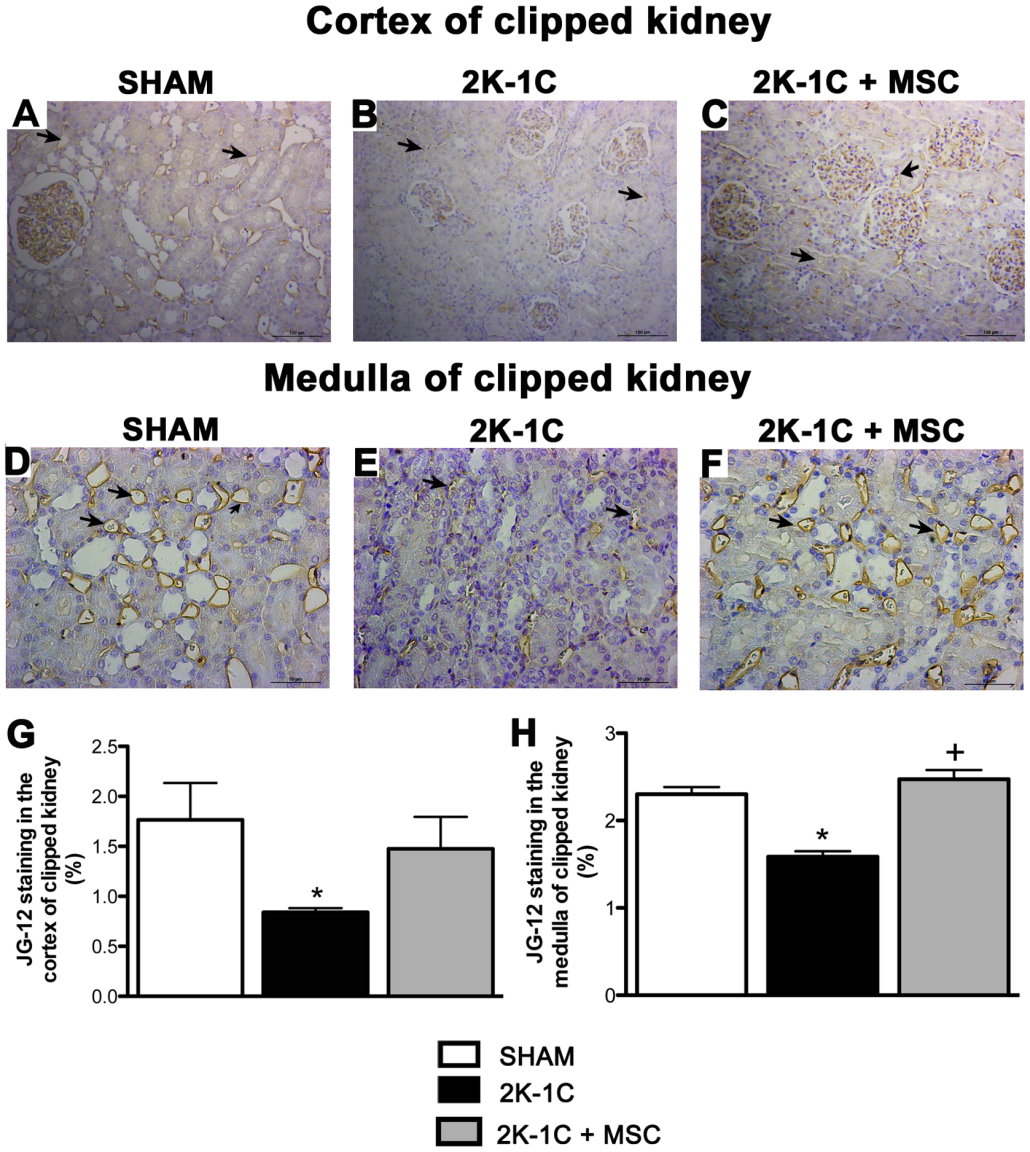
Capillary staining. Representative image of JG-12 staining of a renal cortex (20x) and medulla (40X) of the clipped kidney of a SHAM (**A and D**), 2K-1C (**B and E**) and 2K-1C + MSC animal (**C and F**). Stained areas were measured using the Image Tool program (**G and H**). The arrows show the positive staining in the arterioles. *P<0.05 vs. SHAM. ^+^ *P<0.05 vs. 2K-1C* (One-way ANOVA followed by the Tukey’s post-test).

Glomerular and peritubular capillary density was analyzed by JG-12 immunohistochemical staining, which is specific for the endothelium, and showed a significant decrease in both cortical and medullary capillaries in 2K-1C animals **(** **Figure 4A, 4B, 4D and 4E** **).** MSC treatment improved the microvascular rarefaction of the clipped kidneys in both renal cortex **(** **Figure 4C** **)** and medulla **(** **Figure 4F** **)**. Quantification is shown in **Figure 4G and 4H** **.**

### MSC Treatment Altered the Protein Expression of Components of Renin-Angiotensin (RAS) in the Medulla of Clipped Kidneys

**Figure 5:**
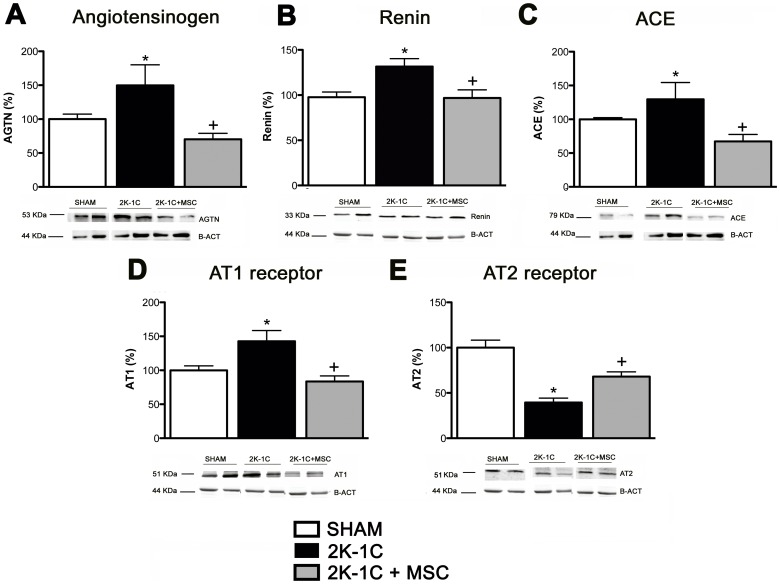
Expression of components of Renin-Angiotensin (RAS) in the medulla of clipped kidneys. Angiotensinogen (**A**), Renin (**B**), ACE (**C**), AT_1_ (**D**) and AT_2_ receptors (**E**) in the medulla of kidney clipped of SHAM (n = 5), 2K-1C (n = 7) and 2K-1C + MSC animals (n = 7). *P<0.05 vs. SHAM. ^+^ *P<0.05 vs. 2K-1C* (One-way ANOVA followed by the Tukey’s post-test).

Six weeks after clamping, the expression of AGTN, ACE and AT_1_ proteins were elevated in the clipped kidneys compared with the SHAM animals (49, 30 and 43%, respectively), and AT_2_ levels were significantly decreased (60%). The expression of all of these proteins were normalized after MSC treatment **(** **Figure 5A-E** **)**.

### Treatment with MSC Leads to Downregulation of Inflammatory Cytokines in the Medulla of Clipped Kidneys

**Figure 6:**
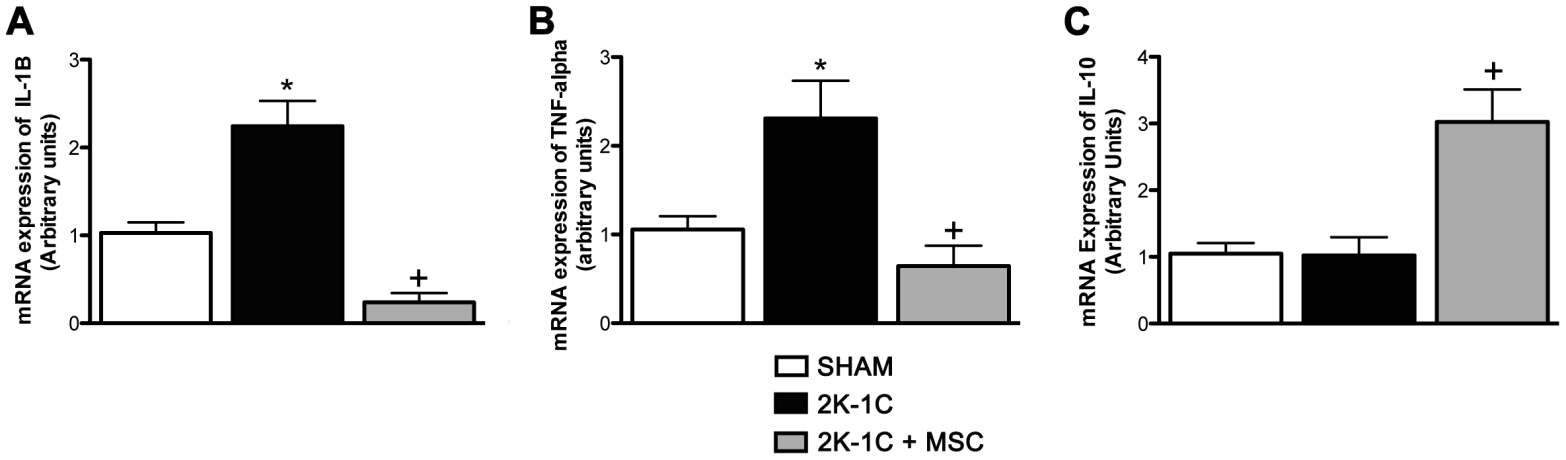
Cytokine mRNA expression. (**A**) IL-1β mRNA expression. (**B**) TNF-α mRNA expression. (**C**) IL-10 mRNA expression. Data expressed as arbitrary units *P<0.05 vs. SHAM. ^+^ *P<0.05 vs. 2K-1C.* (One-way ANOVA followed by the Tukey’s post-test).

qRT-PCR results showed greater expression of the inflammatory cytokines IL-1β and TNF-α in the renal medulla of 2K-1C rats. After the treatment, the expression levels of each of these genes were reduced **(** **Figure 6A-B** **)**. Although the anti-inflammatory cytokine (IL-10) was unchanged in 2K-1C rats, the MSC significantly increase the expression levels of IL-10 in 2K-1C animals **(** **Figure 6C** **)**.

## Discussion

The main results obtained in this study showed that MSC therapy in the 2K-1C model (i) prevented the progressive increase of arterial blood pressure, (ii) improved renal morphology and microvascular rarefaction, (iii) reduced fibrosis, proteinuria and inflammatory cytokines, (iv) suppressed the intrarenal RAS in the medulla of the clipped kidneys, iv) decreased sympathetic hyperactivity in anesthetized animals and v) MSC were detected at the CNS suggesting that the cells crossed the blood-brain barrier.

The bone marrow is the most frequent source of cells used for clinical repair. The bone marrow mononuclear cell fraction not only contains a complex assortment of different stem/progenitor cells, but is also known to migrate to damaged tissues, including ischemic areas [20]. The bone marrow-derived MSC have been enthusiastically investigated for their reparative and regenerative properties since the 1970's [21], [22]. Although in most cases the cells are administered directly into the damaged tissue, in the present study they were systemically administered in order to evaluate whether these cells would reach other potentially damaged organs and tissues including the CNS, a site potentially important in this model of renovascular hypertension [11]. Our results showed that the MSC mainly localized to the clipped kidneys although they were also detected in the medulla oblongata in the CNS. This result corroborates the previous studies, suggesting that the MSC are able to contribute directly to tissue repair, mainly through paracrine and/or endocrine pathways [18]. Moreover, considering that this model of renovascular hypertension is dependent on the activation of SNA and that MSC were found in the medulla oblongata, we evaluated the role of SNA in the antihypertensive effect of MSC. In fact, we have previously observed that the vasomotor sympathetic tone significantly increased after 4^th^ week [12]. The abnormal activity of neurons in the medulla oblongata and the presence of MSC in this region suggest that MSC could cross the blood-brain barrier and in somehow reduce SNA leading to reduction in blood pressure. In fact, the treatment with MSC prevented the progressive increase in SBP in 2K-1C animals. The beneficial effects of this type of cell therapy was successfully performed in others models of hypertension [23], [24], however, the mechanism by which MSC minimized the hypertension is not clear. However, the presence of MSC in the CNS suggests that the effect of this treatment on SBP could be at least in part be due to an interference with SNA leading to reduction of sympathetic activity to the cardiovascular system. Indeed, in our results, we observed that the MSC treatment reduced renal SNA in 2K-1C rats.

On the other hand, it is well established that the activation of RAS induced by reduced renal artery flow is imperative to initiate the increase in SBP, however, the chronic renal hypoperfusion causes renal damage that, in turn, perpetuates the hypertension. Six weeks after the clipping, 2K-1C rats presented with significant proteinuria, indicating that the integrity of the glomerular barrier was lost by the chronic hypoperfusion. MSC treatment significantly reduced proteinuria, suggesting that MSC restored, albeit partially, the selectivity of the glomerular capillary wall.

The renal architecture was severely damaged by chronic ischemia, and it was significantly improved by MSC. Interestingly, the fibrosis marker was higher in the renal medulla when compared with the renal cortex of the clipped kidneys, and this behavior may explain the higher amount of MSC observed in the medulla. In fact, there was an improvement in the morphology of the clipped kidneys primarily in the medulla after MSC treatment, and animals indeed showed less fibrosis when compared with the kidneys of the 2K-1C untreated animals. These partially improved outcomes were also seen following other diseases, such as acute kidney injury [14] and the remnant model kidney [13]. Chade et al (2009) [25] demonstrated that administration of endothelial progenitor cells (EPC) directly in the clipped kidney (2K-1C) at the end of the sixth week after clipping improved renal function by improving vascular remodeling and inducing the expression of angiogenic factors. EPC were renoprotective and attenuated renal dysfunction and damage in chronic atherosclerotic renal artery stenosis and consequently decreased the injury signals [26].

Indeed, previous studies showed that when the kidney is exposed to chronic renal artery stenosis it results in significant functional deterioration presented by renal inflammation, fibrosis, and microvascular rarefaction and remodeling [3], [27]. It is known that intrarenal microvascular disease likely aggravates the progression of renal injury in renovascular hypertension and may account for the renal failure. Our results demonstrated that the density of glomerular and peritubular capillaries was restored after the treatment with MSC in the cortex and medulla of clipped kidneys, indicating that these cells play important role in improving neovascularization improving renal function.

2K-1C animals showed polyuria with a decrease in the net Na^+^ excretion, indicating that the polyuria was not sodium dependent, but was most likely caused by a deficient mechanism of urine concentration. It has been demonstrated that the plasma levels of ADH are not changed in this model of hypertension [28], however, the microvascular rarefaction could hinder ADH in reaching the collecting duct, contributing to an increase in urinary volume. But, in spite of significant improvement of renal vascularization, MSC treatment was not effective to correct this defect. A previous study showed that the expression of AQP 1–3 was significantly decreased in the clipped kidney [29], [30]. Thus, a downregulation of AQPs in the clipped kidney may, in part, account for the increased urinary volume.

It is well established that 2K-1C is a RAS-dependent model, and in addition to the systemic activation of RAS, we found that 2K-1C animals presented with an intrarenal upregulation of the angiotensinogen and AT_1_ receptor and a downregulation of the AT_2_ receptor. We have not determined the intrarenal Ang II content, but these results suggest that the chronic renal hypoperfusion induced an activation of intrarenal RAS that could contribute to renal damage. Moreover, Prieto-Carraquero and cols, in 2008 [31], showed that renal medullary tissue of 2K-1C rats showed greater levels of renin protein, renin mRNA, Ang I, Ang II and renin activity than sham rats. These results suggest that intrarenal RAS contributed to renal inflammation [2] and fibrosis [32]. MSC treatment normalized the expression of RAS components, resulting in less inflammation and fibrosis. The mechanism by which MSC downregulated intrarenal RAS components is not know at this moment, but it is an important issue warranting further investigation because the intrarenal RAS activation plays a pivotal role in many kidney diseases, including diabetic nephropathy.

There is evidence that persistent inflammation contributes to MSC migration to damaged tissue [33]. Our results showed a higher expression of IL-1β and TNF-α mRNAs in the renal medulla of 2K-1C rats, which were significantly reduced in the animals treated with MSC, together with an increase in the expression of the anti-inflammatory IL-10. It is well known that MSC secrete many molecules under inflammatory stimuli. Growing evidence suggests that the mechanisms by which MSC lead to this tissue amelioration may be caused by their ability to undergo to transdifferentiating, fusing, and/or secreting of trophic factors producing the paracrine effects [22], [34].

## Conclusion

Our data support the notion that renal injury promotes a critical signaling mechanism underlying the development of hypertension in the 2K-1C rat model, and MSC treatment can be a promising strategy to treat chronic renal ischemia and renovascular hypertension in the near future.
